# Effect of atorvastatin 1% mouthwash in the prevention of radiotherapy induced mucositis: A pilot study

**DOI:** 10.22088/cjim.13.4.800

**Published:** 2022

**Authors:** Shahram Ala, Majid Saeedi, Arash Ghasemi, Melika Namdari, Neda Koulaeinejad

**Affiliations:** 1 Department of Clinical Pharmacy, Faculty of Pharmacy, Mazandaran University of Medical Sciences, Sari, Iran; 2 Department of Pharmaceutical Sciences, Faculty of Pharmacy, Mazandaran University of Medical Sciences, Sari, Iran; 3 Department of Radiology and Radiation Oncology, Faculty of Medicine, Mazandaran University of Medical Science, Sari, Iran; 4 Student Research Committee, Faculty of Pharmacy, Mazandaran University of Medical Sciences, Sari, Iran; 5 Department of Clinical Pharmacy, Faculty of Pharmacy, Tehran Medical Sciences, Islamic Azad University, Tehran, Iran

**Keywords:** Atorvastatin, Mouthwash, Oral mucositis, Radiotherapy

## Abstract

**Background:**

Oral mucositis is a troublesome symptom for people who receive radiotherapy and chemotherapy and it is a dose-dependent factor. Atorvastatin is a HMG-CoA reductase inhibitors and various studies have proven its anti-inflammatory effects. The goal of this study was to evaluate atorvastatin 1% mouthwash effects in prevention of radiotherapy-induced mucositis.

**Methods:**

Atorvastatin 1% suspension was prepared for mouthwash in this randomized, double-blind clinical trial. Thirty patients randomly received atorvastatin or placebo mouthwash. They had to gargle 5cc of mouthwash, 3 times per day during radiotherapy. The severity and pain of mucositis was evaluated every week, during their treatment.

**Results:**

The severity of mucositis between the two study groups was significant every four weeks (p<0.05) and the percentage of patients with more severe mucositis was less in the atorvastatin group. It is found that the pain intensity was lower after 3 and 4 weeks in atorvastatin group.

**Conclusion:**

These findings indicated that atorvastatin mouthwash showed a significant activity in relieving of radiotherapy-induced oral mucositis and pain.

Each year, many people around the world get cancer. So an approach is required for optimal treatment planning and post-treatment response assessment (1). Radiotherapy is one of the common treatment protocols which may be used alone or in addition to chemotherapy. Some adverse effects such as mucositis may be detected while going through radiotherapy (2). 

Oral mucositis occurs in patients receiving conventional-dose cytotoxic chemotherapy and in those who are prepared with radiation-containing regimens (3). It is an indirect effect of radiotherapy which inhibits the oral epithelium cell mitosis and it is usually exposed 7 to 10 days after radiotherapy inception (4). Mucositis formation appears in 5 phases (5). There is no consensus on the best clinical protocol for the prevention and treatment of mucositis (6). Mucositis can also be associated with complications. For this reason, it is necessary to minimize and prevent it as much as possible (6). Atorvastatin is a HMG-CoA reductase inhibitor which inhibits cholesterol biosynthesis. Another use of atorvastatin is its anti-inflammatory impacts (7-11). 

This study is a first randomized clinical trial that evaluated the effects of atorvastatin 1% mouthwash on patients diagnosed with different types of cancer, who had already undergone radiotherapy treatment.

## Methods

The 1% atorvastatin mouthwash was prepared in the Department of Pharmaceutics, Faculty of Pharmacy using the following materials: the appropriate amounts of atorvastatin powder, glycerin, methyl paraben, PEG 200, xanthan gum, sodium saccharin, tween 80 and distilled water. The content of atorvastatin suspension was determined at 24, 48 and 72 hour and compared with initial content. The placebo was prepared according to the same method, but atorvastatin was not added to the mouthwash. The final preparations were filled in the same bottles and labeled. Both mouthwashes were similar in color, odor and taste.

This randomized, placebo-controlled, double-blind clinical trial was carried out in Radiotherapy Center of Imam Khomeini Educational Hospital of Mazandaran University of Medical Sciences, Sari, Iran. This study was in accordance with Declaration of Helsinki. 

The trial registration code is IRCT201502033014N6. Also, the ethical approval number is IR.MAZUMS.REC.1393.1420. All patients gave written informed consent before enrolment. Also, they were informed that at any time they did not want to continue the trial, we excluded them from the study. 

All patients with cancer, especially with head and neck who experienced radiotherapy-induced mucositis for the first time and were older than 18 years were included in this study. Drug intolerance before first week, incorrect use of mouthwash, receiving oral atorvastatin and anti-inflammatory drugs, history of chemotherapy and development of mucositis (deterioration of mucositis grade during the trial) were the exclusion criteria. Patients received radiotherapy daily for 4 weeks (other than Thursdays and Fridays).

 A Siemens PRIMUS^TM^ linac dual energy machine operating in the 6MVphoton mode was used. The average dose used was 63 Gy. The radiotherapy site was the mouth and throat.

Simple randomization was performed using a table of random numbers. This procedure was carried out by a researcher which he did not attend in subsequent parts of the study. People who went blind in this study included the patients, attending physician and investigators.

Immediately upon starting the radiotherapy sessions, the mouthwash was given to the patients. The patients had to gargle with 5 cc of mouthwash 3 times a day, for at least 5 min, but not to swallow. When the patients referred to Mostafavi Clinic of Mazandaran University of Medical Sciences, they were evaluated by the researcher. 

As described below, WHO scoring was used to assess mucosal severity: Grade 0 (none): None. Grade I (mild): Oral soreness, erythema. Grade II (moderate): Oral erythema, ulcers, solid diet tolerated. Grade III (severe): Oral ulcers, liquid diet only. Grade IV (life-threatening): Oral alimentation impossible (12).

All patients were checked every 7 days during the radiotherapy treatment in terms of mucositis development and changes in oral cavity tissues. The inflammation, erythema, bleeding, infection and liquid and solid swallowing ability were assessed. Also, the pain intensity was evaluated by a visual analogue scale. The highest score 10 representing intolerable pain and 0 showing the absence of pain. The blood tests were also collected to determine serum creatinine and hemoglobin, white blood cells, platelet and blood urea nitrogen at baseline.

## Results

**Figure 1:**
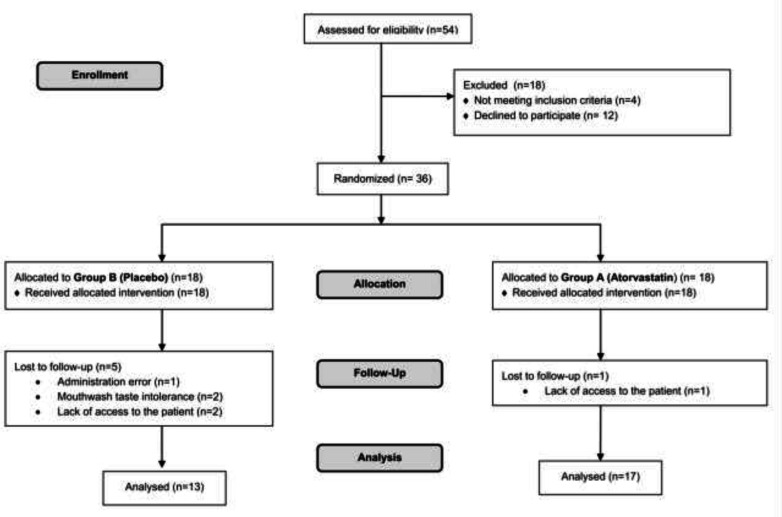
Consort flowchart of the study

**Figure 2:**
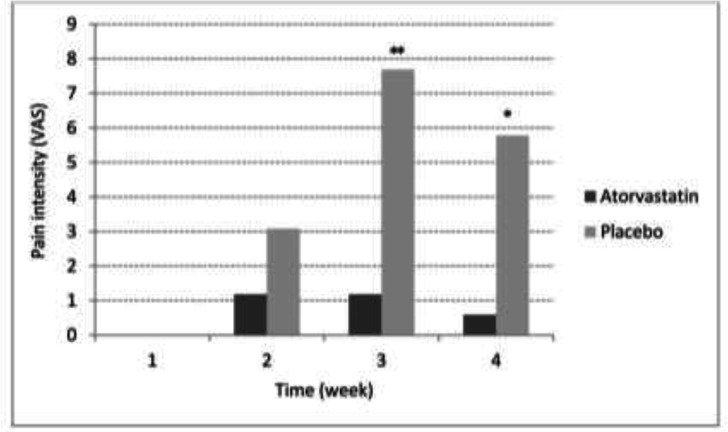
Pain intensity (VAS) on different weeks in atorvastatin mouthwash 1% and placebo groups

As depicted in fig.1, of the 54 eligible patients, 18 patients were not randomized because of the decline to participate and did not have the inclusion criteria. Full details of demographic characteristics, basal hematological test data and distribution of different types of cancers are represented in table 1. According to table 2, there was a significant difference between the two study groups in the whole four weeks. The intensity of mucositis-associated pain are shown in figure 2. 

**Table 1:** Baseline characteristics of patients

**Variables**		**Atorvastatin**	**Placebo**	**P-value**
Number of patients		17	13	
Age (Y), (Mean ± SD)		53.5±15.08	62.53±12.6	0. 4
Sex (Male/Female), n		11/6	4/9	0.07
Laboratory test values (Mean±SD)	WBC (10^3^ /mm^3^) BUN (mg/dl) PLT (10^3^ /mm^3^) Hb (g/dl) SCr (mg/dl)	5.8 ± 1.7 20.4 ± 3.9 230 ± 63 12.14 ± 1.78 1 ± 0.29	9.2 ± 1.0 19.1 ± 3.7 254 ± 98 12.1 ± 1.15 0.8 ± 0.13	0.4 0.5 0.4 0.5 0.6
Type of cancer, N (%)	Tongue s.c.c Hodgkin’s lymphoma Lymphoma Nasopharynx cancer Multiple myeloma Cervical mass Larynx cancer Submandibular lymphoma Esophagus cancer	6 (35.3) 3 (17.6) 1 (5.9) 5 (29.4) 1 (5.9) 1 (5.9) 0 (0) 0 (0) 0 (0)	3 (23.1) 2 (15.4) 1 (7.7) 3 (23.1) 0 (0) 1 (7.7) 1 (7.7) 1 (7.7) 1 (7.7)	0.12

WBC: white blood cells, BUN: blood urea Nitrogen, PLT: platelet, Hb: serum hemoglobin, SCr: serum creatinine

**Table 2:** Severity of mucositis in the two study groups at different time points

**Time (week)**	**Grade**	**Atorvastatin, n (%)**	**Placebo, n (%)**	**P -value**
**1**	0 I II III IV	15 (88.2) 1 (5.9) 1 (5.9) 0 (0) 0 (0)	4 (30.8) 9 (69.2) 0 (0) 0 (0) 0 (0)	0.001
**2**	0 I II III IV	7 (41.0) 9 (52.9) 0 (0) 1 (5.9) 0 (0)	1 (7.7) 11 (84.6) 1 (7.7) 0 (0) 0 (0)	0.01
**3**	0 I II III IV	4 (23.5) 7 (41.2) 0 (0) 5 (29.4) 0 (0)	0 (0) 3 (23.1) 4 (30.8) 6 (46.2) 0 (0)	0.024
**4**	0 I II III IV	1 (5.9) 10 (58.8) 0 (0) 0 (0) 1 (5.9)	0 (0) 1 (7.7) 2 (15.4) 4 (30.8) 0 (0)	0.005

* p<0.05, ** p<0.01

## Discussion

By studying the degree of mucositis in two groups of patients , atorvastatin recipient showed a significant decrease .The frequency of mucositis manifestation in atorvastatin group was significantly less in comparison with placebo group in the total 4 weeks (p<0.05). According to other results farther than mucositis severity, there was a significant difference in pain frequency. Atorvastatin mouthwash reduced pain after third and fourth weeks. One of the theories about this effect is also the inflammation decrease by atorvastatin. As mentioned above, since one of the main mucositis causes is increasing inflammatory markers such as IL-2, IL-6 and etc, anti-inflammatory effects of atorvastatin is justifiable. The results of this study are in agreement with several other studies as follows. In 2018, Özdoğan et al. evaluated the effects of locally administration of atorvastatin (2% w/v) containing chitosan formulations in the treatment of periodontitis in rats. The administration of atorvastatin could decrease the release of pro-inflammatory cytokines. Also, the alveolar bone healing was significant (13).

To the best of our knowledge, this study is the first to evaluate atorvastatin mouthwash in the prevention of radiotherapy-induced mucositis. Nevertheless, our study has few limitations. Patients’ nutritional status was not evaluated. This item can affect developing or haling of mucositis. Small sample size is another limitation of our study. It is suggested that larger studies with severity assessment of other mucositis problems for example pain, be conducted in the future. In conclusion atorvastatin 1% mouthwash could effectively reduce pain, erythema and ultimately, mucositis in comparison with placebo group with significant difference. 
